# Association between the dietary index for gut microbiota and frailty: the mediating role of body mass index

**DOI:** 10.3389/fnut.2025.1573199

**Published:** 2025-07-18

**Authors:** Jiawei Lei, Tingting Feng, Tian Tian, Ziyun Zhuang, Guilei Zhang, Ying Liu, Zhenrong Yang, Yuting Wang, Xin Zhang, Wei Sun, Jiahe Wang

**Affiliations:** ^1^Department of Family Medicine, Shengjing Hospital of China Medical University, Shenyang, China; ^2^Shantou University Medical College, Shantou, China; ^3^Department of Nephrology, Shengjing Hospital of China Medical University, Shenyang, China; ^4^Department of Cardiology, Shengjing Hospital of China Medical University, Shenyang, China; ^5^Department of Infection Diseases, The First Affiliated Hospital of China Medical University, Shenyang, China; ^6^Department of Ultrasound, Shengjing Hospital of China Medical University, Shenyang, China

**Keywords:** diet, gut microbiota, frailty, mediation, body mass index, NHANES

## Abstract

**Background:**

The Dietary Index for Gut Microbiota (DI-GM), a newly introduced metric, indicates gut microbiota diversity. However, its correlation with frailty remains unexplored.

**Method:**

A total of 25,320 individuals were included in the 2007–2020 National Health and Nutrition Examination Survey (NHANES). Dietary recall data were calculated by averaging intake values from two separate 24-h dietary recall interviews. Frailty was assessed using the 49-item frailty index. The relationship between DI-GM and the frailty phenomenon was examined by applying a weighted logistic regression model. A comprehensive sensitivity analysis was undertaken, incorporating restricted cubic splines for modeling non-linear effects, stratified subgroup analyses to explore effect modification, and multiple imputation techniques to address potential missing data concerns.

**Results:**

Higher DI-GM scores and gut microbiota-beneficial dietary components were significantly associated with reduced prevalence of frailty (Frailty Index: OR = 0.987, 95% CI: 0.977–0.997, *P* = 0.014; Frailty: OR = 0.941, 95% CI: 0.902–0.980, *P* = 0.004). Restricted cubic spline analysis revealed a non-linear relationship between DI-GM and frailty. Body Mass Index (BMI) mediated this relationship, accounting for 17.57% of the association.

**Conclusion:**

We concluded that a higher DI-GM score is associated with a lower risk of frailty, partly via BMI mediation. Future research should validate these findings using longitudinal studies.

## Introduction

Frailty is a physical condition caused by the accumulation of age-related deficits, characterized by reduced physiological reserves and loss of resistance to stressors. Frailty is a complex and multi-dimensional concept, which is not limited to physical decline, but also includes cognitive, emotional, social function, and other aspects of decline, the core of which all point to an individual’s increased vulnerability in performing daily activities. Frailty is particularly prevalent among older people and significantly impacts their mobility, daily life activities, and overall quality of life (1). Studies have found that frailty is closely related to a variety of adverse health outcomes (such as falls, hospitalization, disability, death, and dementia) (1, 2). Furthermore, it has been confirmed to be a predictor of mortality, catastrophic health expenditure, postoperative adverse outcomes, and adverse outcomes of chronic diseases (3–7). Moreover, frailty is often accompanied by other chronic conditions, such as diabetes, hypertension, and cardiovascular disease, which further complicate an individual’s health status (8–11). Therefore, identifying and managing frailty is crucial for promoting healthy aging and improving the quality of life among older people.

In addition to frailty’s multifaceted impacts, emerging research has highlighted the significant role of the gut microbiota in human health. The gut microbiota, a complex and diverse community of microorganisms residing in the human gastrointestinal tract, plays a crucial role in various physiological processes, influencing nutrient provision, metabolic processes, antibacterial effects, immune regulation, the brain-gut axis, and cardiovascular health (12–18). Additionally, recent studies have shown that the gut microbiota can influence the human’s response to diet and exercise, affecting weight loss and muscle growth (19) and may potentially slow the aging process and extend lifespan (20). Research indicates a close association between alterations in the gut microbiota and chronic low-grade inflammation. Dysbiosis, or an imbalance in the gut microbiota, can compromise intestinal barrier function, allowing harmful substances such as lipopolysaccharide (LPS) to enter the systemic circulation (21, 22). This chronic inflammation is a core characteristic of frailty, accelerating cellular aging, impairing metabolic function, and diminishing the body’s ability to cope with stressors (23, 24).

Studies suggest that both short-term and long-term dietary habits can alter the gut microbiota’s composi-tion and function, profoundly impacting human health (25, 26). To further explore the connection between diet and gut microbiota, Bezawit E. Kase and colleagues developed the Gut Microbiota Dietary Index (DI-GM) based on a com-prehensive review of 106 studies. The DI-GM is designed to assess the quality of diet concerning maintaining a healthy gut microbiota. It is calculated using data from dietary intake surveys, such as those conducted in the NHANES study, with scores ranging from 0 to 13. Higher scores indicate a more favorable diet for gut microbiota health.

The DI-GM is based on 14 dietary components identified as beneficial or detrimental to gut microbiota. Beneficial components include avocado, broccoli, chickpeas, coffee, cranberries, fermented dairy, fiber, green tea, soy, and whole grains, while detrimental components include red meat, processed meat, refined grains, and high-fat diets. The final DI-GM score reflects the overall balance of these dietary components, with higher scores indicating a healthier diet for gut microbiota. Although previous studies have linked DI-GM to health outcomes such as depression (27) and metabolic syndrome (28, 29), no research has yet explored the relationship between DI-GM and frailty, as well as the mediating role of BMI in this association. To date, there are no studies on the effects of DIGM-mediated BMI on frailty, and our study fills this gap.

This study aims to examine the relationship between DI-GM and frailty using na-tionally representative NHANES data, while also investigating the potential mediating role of BMI in this relationship.

## Materials and methods

### Data sources

The study utilized data acquired from the National Health and Nutrition Examination Survey (NHANES), a valuable cross-sectional survey conducted by the Centers for Disease Control and Prevention (CDC). NHANES employs a nationally representative, complex, multi-stage probability sampling design to comprehensively evaluate the health and nutritional status of adults and children in the United States. The NHANES study protocol received approval from the Research Ethics Review Board of the National Centre for Health Statistics (Continuation of Protocol #2005-06, Protocol #2011-17), Continuation of Protocol #2011-17, Protocol #2018-01 Effective beginning October 26, 2017). All participants provided written informed consent, ensuring compliance with ethical standards and safeguarding participant rights.

### Survey design and population

**FIGURE 1:**
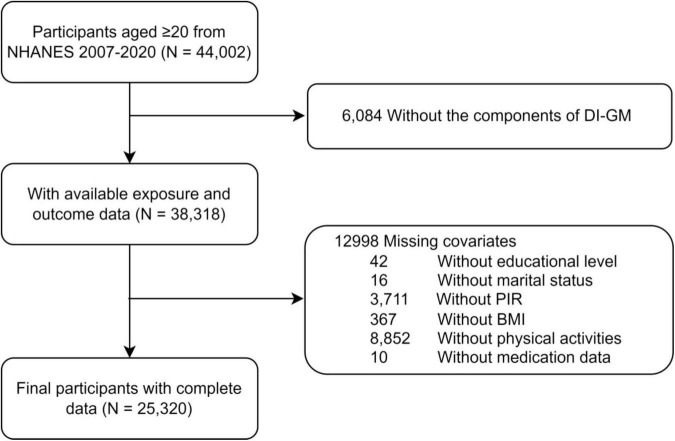
Flowchart of participants of the NHANES 2007–2020.

This study analyzed data from 44,002 participants aged 20 years or older, covering the period from 2007 to March 2020. After excluding participants with missing DI-GM data (*n* = 6,084) or incomplete covariate information, including education level, marital status, PIR, BMI, physical activity and (*n* = 12,998), a total of 25,320 participants met the inclusion criteria for the final analysis. The detailed participant selection process is illustrated in Figure 1.

### The assessment of DI-GM

In the NHANES study, participants underwent two 24-h dietary recall interviews to evaluate their dietary intake. The first interview was conducted in the Mobile Examination Centre (MEC), while the second was completed via telephone, with a time interval of 3–10 days between the two interviews. During these interviews, participants reported all foods and beverages consumed within the past 24 h. Researchers used the food composition database from the USDA Dietary Studies Food and Nutrient Database (FNDDS) to assign codes and quantities to these foods and beverages (30).

Kase et al. (31) studies identified 14 dietary components, including specific foods or nutrients as part of the DI-GM based on a specific scoring criterion. The beneficial components for gut microbiota included avocados, broccoli, chickpeas, coffee, cranberries, fermented dairy products, fiber, soy, green tea, and whole grains. Conversely, the detrimental components included red meat, processed meat, refined grains, and high-fat diets (which comprised ≥ 40% of energy from fat). The DI-GM was calculated based on the average intake from the two 24-h dietary recalls. For beneficial foods, a score of 1 was assigned if the intake exceeded the sex-specific median, and 0 otherwise. For detrimental foods, a score of 0 was assigned if the intake was equal to or exceeded the sex-specific median and 1 otherwise. The final DI-GM score was obtained by summing the scores for all components, with a range of 0–14. A higher DI-GM score indicated a healthier gut microbiota (32). The overall DI-GM score is obtained by summing up the individual scores, ranging from 0 to 14 (including a range of 0–10 for foods beneficial to the gut microbiota and 0–4 for foods detrimental to the gut microbiota). Detailed information is provided in Supplementary Table 2.

### The assessment of frailty

Based on the standard procedures proposed by Searle et al. (33) and his colleagues, we constructed the frailty index (FI) to provide a quantitative measurement of frailty levels. The FI was designed to include traits representing health deficits across multiple domains. In selecting variables, we ensured that they were age-related and spanned multiple domains, including diseases, functional status, and cognition. Variables with either a high incidence (*r* > 0.80) or a strong inter-variable (*r* > 0.95) with others were excluded to minimize redundancy. For each health deficit, values ranging from 0 to 1 were assigned according to their respective severity levels. This approach allowed for the integration of both continuous and categorical variables in our calculation process. The FI score is calculated as the ratio of the total number of health deficits to the total number of variables considered, typically ranging from 0 to 1. A higher FI score signifies greater physical vulnerability.

A frailty index, comprising 49 items, was developed adhering to the standard construction procedure. This index encapsulated a wide array of deficits spanning multiple systems, including chronic diseases, activities of daily living, depressive symptoms, cognitive function, anthropometric measurements, physical performance, general health status, healthcare utilization, as well as laboratory values. The specific deficits incorporated within each system, along with their respective cut-off points, are detailed in Supplementary Table 1. To derive the frailty index score, the number of deficits exhibited by each participant was divided by the total number of considered deficits, yielding a score that ranged from 0 to 1. In the descriptive analysis, a threshold of 0.25 was employed to classify individuals as frail (34).

### Covariates

Based on the specifics of the references and studies, we considered potential confounding variables that could have contributed to frailty, primarily demographic and lifestyle-related questionnaire information. This information was collected using standardized questionnaires during in-home interviews and included gender, age, race/ethnicity, education, marital status, family poverty income ratio (PIR), BMI, physical activity and use of anti-infective prescription medications (35, 36). Race/ethnicity is classified as non-Hispanic White, non-Hispanic Black, Mexican American, other Hispanic, or other race. Education level is divided into high school and below, above high school. Marital status was divided into three categories: never married, married, and widowed/divorced/separated. The Household poverty to Income ratio (PIR) divides household income by the specific poverty guideline for the survey year (37). Physical activity levels are measured by metabolic equivalent (MET) values, and all activities are assigned an intensity level based on a rate of energy consumption expressed as MET, which is obtained by multiplying the time of activity (minutes) and the corresponding metabolic equivalent score (38). Use of anti-infective prescription medications was ascertained based on self-reported data from participants during the interview. The Prescription Medications—Drug Information file from the NHANES database was used to identify and classify these medications. Specifically, this file provides information on the therapeutic drug classes associated with each reported drug and ingredient, allowing us to determine which medications were classified as anti-infectives. This classification was facilitated by the Lexicon Plus^®^ database, a comprehensive database of all prescription and some non-prescription drug products available in the U.S. drug market, developed and maintained by Cerner Multum, Inc. (39, 40).

### Statistical analyses

Following the guidance provided by the NHANES analysis manual, we performed all analyses with consideration for the dietary sampling weights and the complex survey design of NHANES. Continuous variables are presented as mean ± standard error (SE), whereas percentages are used for categorical variables to describe participants’ characteristics. Wilcoxon rank-sum test and chi-square tests were applied to examine the relationships between continuous and categorical variables and frailty, respectively.

Multiple logistic regression models were employed to estimate the adjusted odds ratios (ORs) and their corresponding 95% confidence intervals (CIs) for the associations between DI-GM and its components and frailty index/frailty. In Model 1, no covariates were considered. Model 2 was adjusted for age, sex, race/ethnicity, education, marital status, and poverty income ratio (PIR). Model 3 further adjusted for body mass index (BMI), physical activity metabolic equivalent tasks (MET) and use of anti-infection drugs based on Model 2. To determine whether certain factors alter this association, a stratified analysis was performed to test whether the association between DI-GM and frailty was robust across age groups (≥ 50 years, < 50 years) and gender groups.

To explore the potential non-linear relationship between DI-GM and frailty, a survey-weighted restricted cubic spline (RCS) model was used, setting up four knots to simulate the dose-response relationship between DI-GM scores (including both the beneficial and unfavorable aspects of the DI-GM index) and the frailty index. We also explored the mediating role of BMI in the association between DI-GM and biological age, conducted mediation analysis using the Bootstrap method, and performed 1,000 simulations according to the normal approximation.

Sensitivity analysis includes subgroup analysis and multiple interpolation. Subgroup analyses were used to examine potential effect modifications stratified by age, sex, race/ethnicity, educational attainment, PIR (PIR was divided into three groups: < = 1.30, 1.31–3.50, and > 3.50 (41)), marital status, take anti-infection drugs and NHANES cycles. To mitigate the impact of missing variables on the results, the missing values are interpolated using multiple interpolations via chained equations, resulting in 5 interpolated datasets based on variables in the final statistical model, which is consistent with previous studies. Detailed information on multiple imputations is available in Supplementary methods.

Data were processed and analyzed using R version 4.4.0. Package “survey” (version 4.4.2) was used for survey sample analysis, package “mediation” (version 4.5.0) was utilized for mediation analysis, and package “mice” (version 3.16.0) was used for multivariate imputation. All tests were two-tailed with a test level of α = 0.05.

## Results

### Characteristics of the participants

Table 1 summarizes the characteristics of a representative sample comprising 448.51 million U.S. adults, with an average age of 45.93 years (SE, 0.27). Among this population, approximately 40.63 million individuals were classified as frail. Compared with non-frail individuals, frail participants were generally older, more likely to be male, married or living with a partner, had lower income and education levels, engaged in less intense physical activity, exhibited lower DI-GM scores, and had a higher BMI.

**TABLE 1:** Baseline of participants of the NHANES 2007–2020.

Variable	Total	Non-frailty	Frailty	*P*-value
Weighted population, n (in millions)	448.51	407.87	40.63	
Age, mean (SE), y	45.93 (0.27)	44.82 (0.26)	57.12 (0.49)	< 0.0001
Age group (n,%)				< 0.0001
> = 50	13,931 (58.00)	13,151 (60.93)	780 (28.60)	
< 50	11,389 (42.00)	9,223 (39.07)	2,166 (71.40)	
Sex (n,%)				<0.0001
Female	12,198 (48.93)	10,560 (47.96)	1,638 (58.70)	
Male	13,122 (51.07)	11,814 (52.04)	1,308 (41.30)	
Race/Ethnicity (n,%)b				<0.0001
Mexican American	3,378 (8.06)	3,086 (8.35)	292 (5.12)	
Non-Hispanic Black	5,424 (10.34)	4,615 (9.86)	809 (15.13)	
Non-Hispanic White	10,862 (67.51)	9,571 (67.65)	1,291 (66.11)	
Other Hispanic	2,394 (5.63)	2,116 (5.60)	278 (5.97)	
Other	3,262 (8.46)	2,986 (8.54)	276 (7.67)	
Education (n,%)				<0.0001
Above high school	14,801 (64.84)	13,462 (66.37)	1,339 (49.50)	
High school and below	10,519 (35.16)	8,912 (33.63)	1,607 (50.50)	
Marital status (n,%)				<0.0001
Divorced/separated/widowed	5,008 (16.65)	3,965 (15.13)	1,043 (31.91)	
Married/living with partner	15,178 (62.66)	13,709 (63.54)	1,469 (53.85)	
Never married	5,134 (20.69)	4,700 (21.33)	434 (14.25)	
PIR, mean (SE)	3.12 (3.24)	3.19 (3.24)	2.36 (5.34)	<0.0001
BMI, mean (SE), kg/m^2^	28.91 (0.09)	28.61 (0.09)	31.98 (0.23)	<0.0001
PAtotal MET, mean (SE)	5,127.16 (91.64)	5,240.92 (99.80)	3,985.38 (157.58)	<0.0001
Take anti-infectives drugs (n,%)				<0.0001
No	23968 (93.98)	21313 (94.41)	2655 (89.58)	
Yes	1352 (6.02)	1061 (5.59)	291 (10.42)	
DI_GM, mean (SE)	4.73 (0.02)	4.75 (0.03)	4.55 (0.05)	<0.0001
Beneficial to gut microbiota, mean (SE)	2.18 (0.02)	2.20 (0.02)	1.94 (0.04)	<0.0001
Unfavorable to gut microbiota, mean (SE)	2.56 (0.01)	2.55 (0.01)	2.61 (0.03)	0.03

BMI, body mass index; PA, physical activity; PIR, poverty income ratio; SE, standard error. All means and SEs for continuous variables and numbers and percentages for categorical variables were weighted. ^a^Includes multi-racial participants. NHANES does not provide a detailed list of all races and ethnicities. ^b^The other category includes all Hispanics, regardless of race, who were not Mexican-American and also includes all non-Hispanics from racial groups other than White or Black.

### Associations between DI-GM and frailty

As presented in Table 2, for every 1-point increase in DI-GM, the prevalence of frailty decreased by 1.5% (OR = 0.985, 95%CI: 0.976, 0.995, *P* < 0.01), and the score of frailty index decreased 0.074 (OR = 0.926, 95%CI: 0.893, 0.960, *P* = 0.001). After adjusting for covariates sex, age, race/ethnicity, education level, marital, and PIR, the above association remained significant in Model 2 (frailty: OR = 0.926, 95%CI: 0.893, 0.960, *P* = 0.001; frailty index: OR = 0.985, 95%CI: 0.971, 0.990, *P* = 0.001). In fully adjusted model 3, DI-GM scores were significantly associated with reduced risk of frailty (frailty: OR = 0.941, 95%CI: 0.902, 0.980, *P* = 0.004; frailty index: OR = 0.987, 95%CI: 0.977,0.997, *P* = 0.014).

**TABLE 2:** Weighted multifactor logistic regression analysis for associations between DIGM and frailty index.

Variables	Outcomes	Model1^a^		Model2^b^		Model3^c^	
		OR (95%CI)	*P*	OR (95%CI)	*P*	OR (95%CI)	*P*
DI_GM	Frailty index	0.985 (0.976,0.995)	0.005	0.981 (0.971,0.990)	0.001	0.987 (0.977,0.997)	0.014
Beneficial to gut microbiota	Frailty index	0.968 (0.956,0.980)	0.001	0.973 (0.962,0.984)	0.001	0.976 (0.965,0.987)	0.001
Unfavorable to gut microbiota	Frailty index	1.018 (1.005,1.032)	0.008	0.997 (0.986,1.009)	0.650	1.008 (0.996,1.020)	0.183
DI_GM	Frailty^d^	0.926 (0.893,0.960)	0.001	0.920 (0.884,0.957)	0.001	0.941 (0.902,0.980)	0.004

OR, Odds Ratio; CI, Confidence Interval; DIGM, Dietary Index of Gut Microbiota.

^a^Model1: The crude model without adjustment for covariates.

^b^Model2: Adjust for sex, age, race/ethnicity, education level, marital, PIR;

^c^Model3: Adjust for model 2, additionally adjusted for sex, age, race, education, marital, PIR, BMI, physical activity total MET, take anti-infectives drugs.

^d^Frailty determines whether the Frailty index is ≥ 0.25.

Table 3 further illustrates the associations between DI-GM and frailty by the survey-weighted logistic regression models. In Model 1, DI-GM was inversely associated with frailty (adjusted OR = 0.926, 95%CI: 0.893, 0.960, *P* < 0.0001). This negative relationship persisted in Model 2 (adjusted OR = 0.920, 95% CI: 0.884, 0.957, *P* < 0.0001) and Model 3 (adjusted OR = 0.941, 95% CI: 0.902, 0.980, *P* = 0.004) after adjusting for potential confounders.

**TABLE 3:** Association between DIGM and frailty, with results weighted for sampling strategy.

Subgroup	Model 1^a^	*P*-value	Model 2^b^	*P*-value	Model 3^c^	*P*-value
**Total population**						
Frailty index[β(95%CI)]	0.985 (0.976,0.995)	0.005	0.981 (0.971,0.990)	<0.001	0.987 (0.977,0.997)	0.014
Frailty^d^ [OR (95%CI)]	0.926 (0.893,0.960)	<0.0001	0.920 (0.884,0.957)	<0.0001	0.941 (0.902,0.980)	0.004
**Female**						
Frailty index[β(95%CI)]	0.967 (0.954,0.980)	< 0.0001	0.977 (0.965,0.990)	< 0.001	0.987 (0.974,0.999)	0.035
Frailty [OR (95%CI)]	0.900 (0.854,0.948)	< 0.001	0.927 (0.878,0.979)	0.007	0.960 (0.908,1.015)	0.152
**Male**						
Frailty index[β(95%CI)]	0.999 (0.986,1.012)	0.999	0.985 (0.973,0.997)	0.014	0.988 (0.975,1.000)	0.057
Frailty [OR (95%CI)]	0.942 (0.892,0.995)	0.032	0.910 (0.854,0.969)	0.004	0.916 (0.860,0.976)	0.007
**Age≥ 50**						
Frailty index[β(95%CI)]	0.960 (0.947,0.974)	<0.0001	0.977 (0.963,0.991)	0.001	0.985 (0.972,0.999)	0.035
Frailty [OR (95%CI)]	0.887 (0.845,0.930)	<0.0001	0.937 (0.892,0.984)	0.010	0.958 (0.910,1.008)	0.097
**Age < 50**						
Frailty index[β(95%CI)]	0.982 (0.972,0.993)	<0.001	0.984 (0.974,0.993)	<0.001	0.989 (0.979,0.999)	0.037
Frailty [OR (95%CI)]	0.867 (0.823,0.913)	<0.001	0.865 (0.816,0.917)	<0.0001	0.881 (0.829,0.936)	<0.0001

OR, Odds Ratio; 95%CI, 95% Confidence Interval.

^a^Model 1 was the crude model without adjustment for covariates.

^b^Model 2 was adjusted for age, sex, race/ethnicity, PIR, and education level.

^c^Model 3 was adjusted as for model 2, additionally adjusted for physical activity total MET, BMI, take anti-infectives drugs.

^d^Frailty determines whether the Frailty index is ≥ 0.25. 95%CI, 95% Confidence Interval. Model adjusted for age, sex, race, education, PIR, PA total MET, take anti-infectives drugs.

**FIGURE 2:**
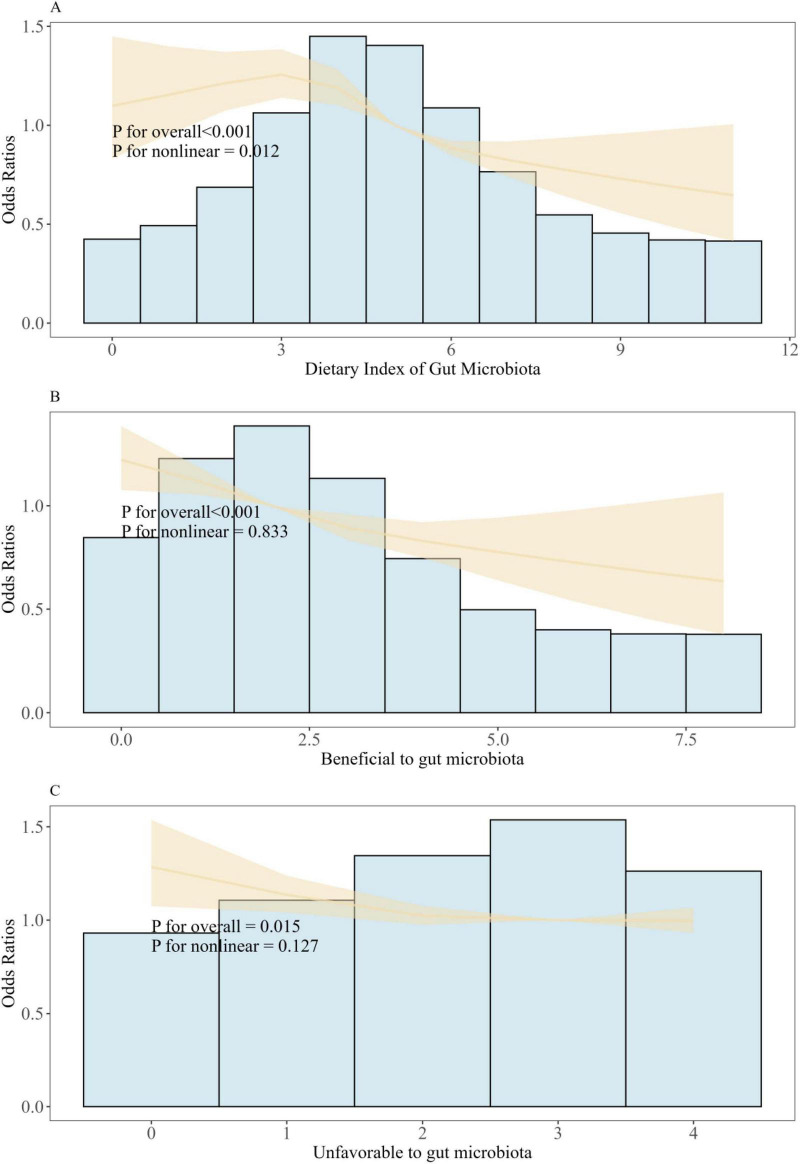
Association between DI-GM and frailty in NHANES 2007–2020 participants by RCS. **(A)** Restricted spline regression showed non-liner association between DI_GM and frailty. **(B)** Restricted spline regression showed liner association between Beneficial to gut microbiota and frailty. **(C)** Restricted spline regression showed liner association between Unfavorable to gut microbiota and frailty. Model adjusted for age, sex, race, education, BMI, PIR, PA total MET, take anti-infectives drugs. Frailty determines whether the Frailty index is ≥ 0.25.

The non-linear relationship between DI-GM and frailty was further explored using restricted cubic splines (RCS) regression. RCS analysis revealed a non-linear association between DI-GM and frailty (*P* for overall < 0.001, *P* for non-linearity = 0.012). However, the effects of both beneficial (*P* for overall < 0.001, *P* for non-linearity = 0.833) and unfavorable (*P* for overall = 0.015, *P* for non-linearity = 0.127) gut microbiota composition on frailty were found to be linear in nature (Figure 2). The threshold effect analysis revealed that 3 was a critical inflection point. When DI-GM was less than 3, the correlation between the two variables was not statistically significant (*P* = 0.472). When DI-GM was greater than 3, a significant correlation existed between DI-GM and the incidence of frailty (*P* < 0.001; Supplementary Table 4).

### Subgroup and sensitivity analyses

**FIGURE 3:**
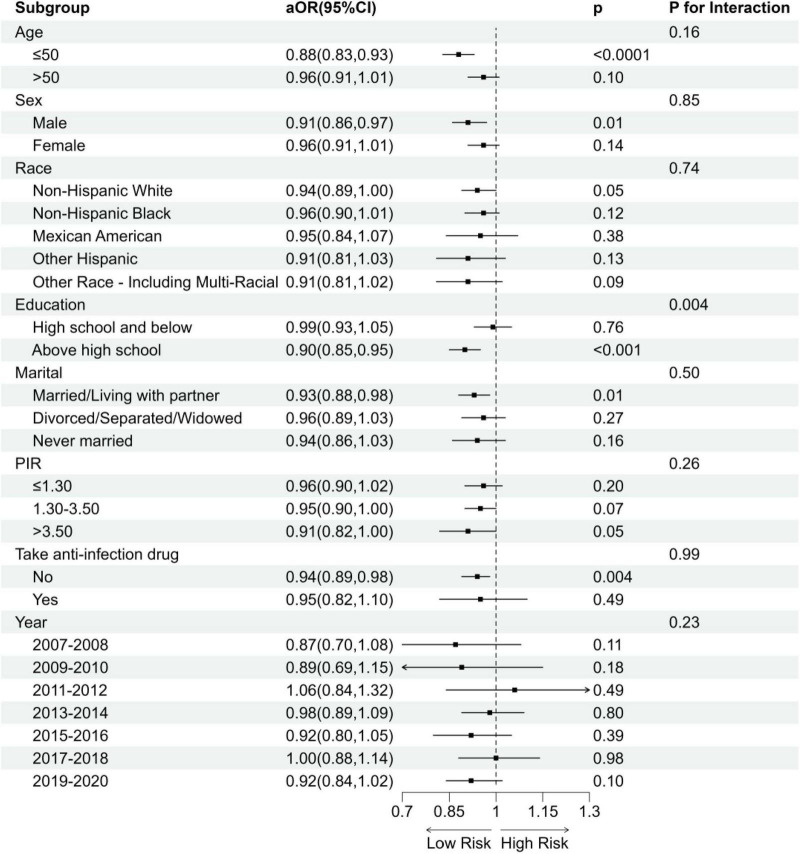
Subgroup analysis of the association between DI-GM and frailty status. aOR, adjusted Odds Ratio; 95%CI, 95% Confidence Interval. Model adjusted for age, sex, race, education, BMI, PIR, PA total MET, take anti-infectives drugs.

To derive more comprehensive results on trend and interaction analysis, this study performed subgroup analyses to investigate whether the relationship between DI-GM and frailty status was influenced by factors such as age, sex, ethnicity, education level, PIR and use of anti-infection drugs, and NHANES cycles (Figure 3). Except for the subgroup of educational level (P for interaction < 0.05), no significant interactions were found.

To handle missing data, we applied multiple imputations by chained equations (MICE), generating five imputed datasets. Multivariate logistic regression analyses on these imputed datasets yielded results consistent with the primary analysis, further supporting the protective role of higher DI-GM scores against frailty. These findings showed similar effect sizes and directions as the original dataset, reinforcing the robustness of our results. Detailed results of the multiple imputation analyses are available in Supplementary Table 3.

### Mediation analysis

**FIGURE 4:**
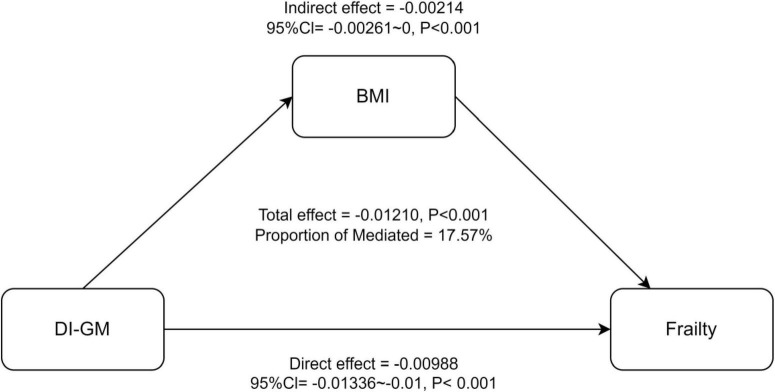
Mediation analysis of BMI in the association between DI-GM and Frailty. 95%CI, 95% Confidence Interval. Model adjusted for age, sex, race, education, PIR, PA total MET, take anti-infectives drugs.

Figure 4 presents the results of the mediation analysis, with adjustments for potential confounders. The total effect of DI-GM on frailty was −0.01210 (*P* < 0.001), while the indirect effect mediated by BMI was −0.00213 (95% CI: −0.00214 to 0, *P* < 0.001). The proportion of the association mediated by BMI was 17.57% (*P* < 0.001).

## Discussion

In our study, we demonstrated for the first time that DI-GM score was significantly negatively associated with frailty (frailty: OR = 0.941, 95% CI: 0.902, 0.980; > 0.25 frailty index: OR = 0.987, 95% CI: 0.977, 0.997). This association remained robust after adjusting multiple covariates. RCS analysis indicated that there is a non-linear relationship between DI-GM and frailty (*P* for non-linearity = 0.012). Notably, both beneficial gut microbiota (*P* for non-linearity = 0.833) and unfavorable gut microbiota (*P* for non-linearity = 0.127) exhibited linear correlations with frailty. Moreover, BMI was identified as a significant mediator of the association between them (OR = −0.00214, 95% CI: −0.00261, 0, *P* < 0.001), with 17.57% of the association being mediated. (42–45)Diet plays a critical role in shaping gut microbiota diversity and function, thereby influencing frailty risk. Adherence to dietary patterns such as the alternative Mediterranean diet (aMED), Recommended Food Score (RFS), Dietary Approaches to Stop Hypertension (DASH), and Mediterranean DASH and Neurodegenerative Delay Intervention (MIND) diet have been associated with reduced frailty risk (42). These diets are characterized by high intakes of fruit, vegetables, whole grains, lean protein, and healthy fats, and low in processed foods and refined sugars. These dietary components positively influence the gut microbiota, potentially reducing inflammation and oxidative stress (42). Specifically, prebiotics, as beneficial components of gut microbiota, can lower inflammatory markers (e.g., CRP, IL-7), enhance antioxidant enzyme activity (e.g., SOD), and mitigate damage from free radicals (44). Furthermore, elevated levels of certain metabolites, such as methionine, histidine, and alanine, are linked to frailty prevention, as they align with metabolic profiles observed in non-frail individuals (45). These findings suggest that specific dietary components can modulate gut microbiota composition and function, influencing metabolic pathways and reducing the risk of frailty.

Mechanistically, the association between DI-GM and frailty can be attributed to immune-inflammatory activation, oxidative stress imbalance, and abnormal amino acid metabolism (46–48). It is reported that the breakdown of antioxidant enzyme activity, such as SOD-1 is universal in frail individuals (46). The amino acid metabolism disorder such as the elevated levels of 3-methylhistidine, alanine, arginine, ethanolamine, and glutamate is associated with muscle loss and functional decline (47). Biomarkers like IL-6, cathepsin S, cystatin C, and GP-acetyl have demonstrated significant associations with frailty index scores in cross-sectional studies (47). Additionally, an 8-year follow-up study revealed that participants with higher baseline hs-CRP levels exhibited a significant increase in frailty index scores over time (48).

Rashidah et al. (49) reported reduced gut microbiota diversity in frail individuals, consistent with our findings (49). Mendelian randomization analysis provides further evidence of a causal relationship between specific gut microbiota genera and frailty (50). Notably, gut microbiota metabolites, such as phenylacetylglutamine (PAGln), can accelerate cellular aging by activating the ADR-AMPK signaling pathway, leading to mitochondrial dysfunction and DNA damage (20). These findings collectively highlight the crucial role of gut microbiota in the pathophysiology of frailty, particularly through mechanisms involving inflammation, oxidative stress, and metabolic dysregulation (51–56). BMI plays a crucial mediating role in the relationship between DI-GM and frailty, highlighting the complex interplay between obesity and frailty (56). While obesity is often associated with a higher risk of frailty, the relationship is not straightforward. Some studies have reported a U-shaped relationship, where both high and low BMI are linked to greater frailty risk (53). This phenomenon, sometimes referred to as the “obesity paradox”, suggests that higher BMI may mask underlying frailty and provide protective effects on muscle mass and bone density (54). Conversely, sarcopenia and other age-related changes may elevate frailty risk in individuals with normal BMI. Abdominal obesity, characterized by a high waist circumference, combined with a non-obese BMI (< 30 kg/m^2^), has been identified as a significant risk factor for frailty (55). Recent studies also highlight the mediating role of BMI in the relationship between gut microbiota-related dietary indices and biological age, consistent with our findings (56). These findings suggest that BMI may influence frailty through multiple mechanisms, including inflammation, metabolic dysfunction, and muscle mass regulation. Further research is needed to disentangle the complex relationship between BMI, gut microbiota, and frailty.

This study has several strengths. Firstly, it utilized the NHANES database, a national representative stratified multi-stage probability survey with extensive and comprehensive data. The use of stratified, multi-stage probability sampling ensures that the subjects represent the population distribution and characteristics of the entire United States. Secondly, the rigorous data collection protocols and quality control measures employed by NHANES enhance the reliability and validity of the findings. Additionally, the sensitivity analyses, including subgroup analyses and multiple imputation, further strengthen the robustness of the results. Finally, this study is the first one to comprehensively investigate the relationship between the DI-GM dietary quality index and frailty, as well as the mediating role of BMI, in a large, diverse population.

The clinical implications of our findings regarding BMI and DI-GM are significant. Regarding BMI, our results suggest that it may not be an independent predictor of frailty, but rather a mediating factor influenced by gut microbiota composition and dietary patterns. Therefore, BMI should be considered as a complementary factor in a comprehensive assessment of frailty risk, alongside other indicators such as physical performance, nutritional status, and chronic disease burden. The DI-GM score, on the other hand, represents a dietary quality index specifically designed to reflect the impact of diet on gut microbiota health. Our findings indicate that the DI-GM score may be a useful tool for identifying individuals at higher risk of frailty and guiding dietary interventions to improve gut microbiota health and potentially reduce frailty risk.

The study also had some limitations. First, it is a cross-sectional study, which cannot prove the causal relationship between DI-GM and frailty. Second, despite efforts to adjust many potential confounders, it is not possible to eliminate residual confounders (e.g., medical conditions, diet, occupation, drug use, other environmental chemicals) and unexpected factors (e.g., genetic influences). Despite these limitations, the study successfully demonstrated the association between DI-GM and frailty, underscoring the necessity for multicenter prospective cohort studies to further investigate this relationship.

## Conclusion

Our study proposed DI-GM, which reflects a diet quality index related to gut microbiota diversity, was found to be inversely associated with frailty prevalence and frailty index. Mediation analysis further explored the mediating role of BMI. Given the close link between diet, gut microbiota, and frailty, further research and dietary interventions for frailty patients will be critical to reducing the prevalence of this disease.

## Data Availability

Publicly available datasets were analyzed in this study. This data can be found at: https://www.cdc.gov/nchs/nhanes/; https://www.cdc.gov/nchs/nhanes/index.htm.
