# Supplementary material for: Association between the dietary index for gut microbiota and frailty: the mediating role of body mass index
Source: Front Nutr. 2025 Jul 18;12:1573199. doi: 10.3389/fnut.2025.1573199 (PMC12313513; doi:10.3389/fnut.2025.1573199)
Supplement: Supplementary file 1 [file Table_1.docx]

**Supplementary Materials Files**

**To: The Mediating Role of Body Mass Index in the Association of the dietary index for gut microbiota and frailty: NHANES 2007-2020**

**Supplementary Methods**

***Dietary Index for Gut Microbiota***

The final DI-GM score is expressed as:

$$\mathrm{DI}-\mathrm{GM}=\sum_{i=1}^{10} B_{i}+\sum_{j=1}^{4} \left( 1-D_{j} \right)$$

where $ B_i $ are beneficial components, and $ D_j $ are detrimental components.

An example: a participant consuming above-median beneficial foods (5 points) and below-median detrimental foods (3 points) would achieve a DI-GM score of $ 5 + (4 - 3) = 6 $.

***Frailty Index***

Each deficit was scored between 0 and 1, with the FI calculated as the ratio of present deficits to total possible deficits:

$$FI=\frac{\text{Number of Deficits Present}}{\text{Total Deficits Assessed}}$$

***Multiple imputation***

Multiple imputation is a widely adopted statistical approach for addressing missing data, commonly applied in fields such as epidemiology, social sciences, and clinical research. This method involves generating multiple plausible datasets through imputation, performing independent analyses on each dataset, and then integrating the results to obtain valid estimates and measures of uncertainty [2, 3]. By doing so, it minimizes potential biases and enhances the representativeness of the findings.

In this study, we utilized the multiple imputation by chained equations (MICE) method, a versatile approach that imputes missing values for each variable sequentially, based on regression models conditioned on observed values from other variables [2, 3]. Five imputed datasets were generated, and the final results were aggregated using Rubin’s Rules, ensuring the reliability and robustness of our conclusions.

1. Kase BE, Liese AD, Zhang J, Murphy EA, Zhao L, Steck SE. The Development and Evaluation of a Literature-Based Dietary Index for Gut Microbiota. Nutrients. 2024 Apr 3;16(7):1045.

2. White IR, Royston P, Wood AM (2011) Multiple imputation using chained equations: Issues and guidance for practice. Stat Med 30:377–399. https://doi.org/10.1002/sim.4067

3. Beesley LJ, Bondarenko I, Elliot MR, et al (2021) Multiple imputation with missing data indicators. Stat Methods Med Res 30:2685–2700. https://doi.org/10.1177/09622802211047346

**Supplementary Table**

**Supplementary Table 1. Variables in the 49-Item Frailty Index and Their Respective Scorings**

**Supplementary Table 2. Variables in the Dietary Index for Gut Microbiota and Their Respective Scorings**

**Supplementary Table 3. Association between DI-GM and Frailty of the NHANES 2007-2020 March participants after multiple imputations.**

**Supplementary Table 1. Variables in the 49-Item Frailty Index and Their Respective Scorings**

| **Item** | **variable** | **code** |
| --- | --- | --- |
| **Cognition** | - | - |
| 1. experience confusion/memory problems | pfq056,pfq057 | yes=1; no=0 |
| **Dependence** | - | - |
| 2. managing money difficulty | pfq060a,pfq061a | no difficulty=0; some difficulty=0.33;  much difficulty=0.66; unable to do=1 |
| 3. walking for a quarter mile difficulty | pfq060b,pfq061b | the same to above |
| 4. walking up ten steps difficulty | pfq060c,pfq061c | the same to above |
| 5. stooping, crouching, kneeling difficulty | pfq060d,pfq061d | the same to above |
| 6. lifting or carrying difficulty | pfq060e,pfq061e | the same to above |
| 7. house chore difficulty | pfq060f,pfq061f | the same to above |
| 8. preparing meals difficulty | pfq060g,pfq061g | the same to above |
| 9. standingup from armless chair difficulty | pfq060i,pfq061i | the same to above |
| 10. getting in and out of bed difficulty | pfq060j,pfq061j | the same to above |
| 11. using fork, knife, drinking from cup difficulty | pfq060k,pfq061k | the same to above |
| 12. dressing yourself difficulty | pfq060l,pfq061l | the same to above |
| 13. standing for long periods difficulty | pfq060m,pfq061m | the same to above |
| 14. grasp/holding small objects difficulty | pfq060p,pfq061p | the same to above |
| 15. attending social event difficulty | pfq060r,pfq061r | the same to above |
| 16. leisure activity at home difficulty | pfq060s,pfq061s | the same to above |
| 17. push or pull large objects difficulty | pfq061t | the same to above |
| **Depressive Symptoms** | - | - |

| **Item** | **variable** | **code** | |
| --- | --- | --- | --- |
| 18. have little interest in doing things | ciqd008,ciqd009,dpq010 | | **~2003**  every day,nearly every day = 1  most days = 0.75  about half the days = 0.50 less than half the days = 0.25 **2005~**  nearly every day = 1 more than half the days = 0.66  several days = 0.33 |
| 19. feeling down, depressed, or hopeless | dpq020,ciqd001,ciqd002 | | the same to above |
| 20. trouble sleeping or sleeping too much | dpq030,ciqd025,ciqd026 | | **~2003**  every night = 1  nearly every night = 0.66 less often = 0.33  **2005~**  nearly every day = 1 more than half the days = 0.66  several days = 0.33 |
| 21. feeling tired or having little energy | dpq040 | | nearly every day = 1 more than half the days = 0.66  several days = 0.33 |
| 22. poor appetite or overeating | ciqd019,ciqd022,dpq050 | | **~2003**  yes = 1  no = 0  **2005~**  the same to above |
| 23. feeling bad about yourself | dpq060,ciqd029 | | the same to above |
| 24. trouble concentrating on things | dpq070,ciqd043 | | the same to above |
| **Comorbidities** | - | | - |
| 25. doctor ever said you had arthritis | mcq160a | | yes = 1; no = 0 |
| 26. ever told you had thyroid problem | mcq160i,mcd160m,mcq160m | | the same to above |
| 27. ever told you had chronic bronchitis | mcq160k,mcq160p | | the same to above |
| 28. ever told you had cancer or malignancy | mcq220 | | the same to above |

| **Item** | **variable** | | **code** | |
| --- | --- | --- | --- | --- |
| 29. ever told had congestive heart failure | | mcq160b | | the same to above |
| 30. ever told you had coronary heart disease | | mcq160c | | the same to above |
| 31. ever told you had angina/angina pectoris | | mcq160d | | the same to above |
| 32. ever told you had heart attack | | mcq160e | | the same to above |
| 33. ever told you had a stroke | | mcq160f | | the same to above |
| 34. ever told you had high blood pressure | | bpq020 | | the same to above |
| 35. doctor told you have diabetes | | diq010 | | yes = 1; no =0; borderline=0.5 |
| 36. ever told you had weak/failing kidneys | | kiq020,kiq022 | | yes = 1; no =0 |
| 37. urine leakage bother you? | | kiq040,kiq050 | | **1999**  yes = 1 ; no = 0  **2001~**  greatly = 1  very much = 0.75 somewhat = 0.5 only a little = 0.25 |
| **Hospital Utilization and Access to Care** | | - | | - |
| 38. general health condition | | huq010 | | excellent,very good,good = 0 fair, poor = 1 |
| 39. health now compared with 1 year ago | | huq020 | | about the same, better = 0 worse = 1 |
| 40. overnight hospital patient in last year | | huq070,hud070,huq071 | | yes = 1, no = 0 |
| 41. times receive healthcare over past year | | huq050,huq051 | | none = 0; 1-4 = 0.5; >=5 =1 |
| 42. number of prescription medicines taken | | rxd030,rxduse,rxd295,rxdcount | | no = 0; 1-4 = 0.5; >=5 =1 |
| **Physical Performance and Anthropometry** | | - | | - |
| 43. body mass index (kg/m^2) | | bmxbmi | | <18.5, ≥30 = 1  25–<30 = 0.5  18.5–25 = 0 |
| **Laboratory Values** | | - | | - |
| 44. glycohemoglobin(%) | | lbxgh | | 0%–5.7% = 0, >5.7% = 1 |

| **Item** | | **variable** | | | **code** | |
| --- | --- | --- | --- | --- | --- | --- |
| 45. red blood cell count (million cells/ul) | | | lbxrbcsi | | | M: 4.7–6.1 = 0, Other = 1  F: 4.2–5.4 = 0, Other = 1 |
| 46. hemoglobin (g/dl) | lbxhgb | | | M: 13.5–18 = 0, Other = 1  F: 12–16 = 0, Other = 1 | | |
| 47. red cell distribution width (%) | lbxrdw | | | 11.6–14.6 = 0, Other = 1 | | |
| 48. lymphocyte percent (%) | lbxlypct | | | 20–40 = 0, Other = 1 | | |
| 49. segmented neutrophils percent (%) | lbxnepct | | | 40–80 = 0, Other = 1 | | |
|  |  | | |  | | |

**Supplement****ary Table 2.** **Components in the Dietary Index for Gut Microbiota and Their Respective Scorings**

| **Components** | **Included Foods** | **Scoring Criteria:** |
| --- | --- | --- |
| Avocados | Avocados | Beneficial to gut microbiota：  For each component, a score of 1 is given if the consumption is at or above the sex-specific median, otherwise, it is 0. |
| Broccoli | Broccoli |  |
| Chickpeas | Chickpeas |  |
| Coffee | Coffee |  |
| Cranberries | Cranberries |  |
| Fermented dairy products | Yogurt, Cheese, Kefir, Sour Cream, Buttermilk |  |
| Fiber | Not applicable |  |
| Green tea | Green tea |  |
| Soybeans | Including soy products like soy milk and tofu |  |
| Whole grains | Whole wheat grain, including bran, germ, and endosperm |  |
| High-fat diet  (% energy from fat) | Not applicable | Unfavorable to gut microbiota：  For a high-fat diet, a score of 0 is given if 40% or more of the energy comes from fat, otherwise, it is 1.  For each of the remaining components, a score of 0 is given if the consumption is at or above the sex-specific median, otherwise, it is 1. |
| Processed meat | Including products like sausages, salami, bacon, and lunch meats made from beef, pork, or poultry |  |
| Red meat | Beef, veal, pork, lamb, and game meats; does not include organ meats or preserved meats |  |
| Refined grains | Grains that do not contain the entire grain substance |  |

**Supplementary Table 3. Association between DI-GM and Frailty of the NHANES 2007-2020 March participants after multiple imputations.**

| **Characteristics** | **Frailty** | | | | **Frailty score** | | | |
| --- | --- | --- | --- | --- | --- | --- | --- | --- |
|  | **Crude model** | | **Adjusted model** | | **Crude model** | | **Adjusted model** | |
|  | **OR (95%CI)** | ***P* value** | **OR (95%CI)** | ***P* value** | **β (95%CI)** | ***P* value** | **β (95%CI)** | ***P* value** |
| DI-GM | 0.921 (0.901, 0.943) | <0.001 | 0.962 (0.940, 0.985) | 0.001 | -0.030(-0.038, -0.022) | <0.001 | -0.012 (-0.019, -0.005) | <0.001 |
| Beneficial to gut microbiota | 0.826 (0.802, 0.850) | <0.001 | 0.874 (0.849, 0.899) | <0.001 | -0.072 (-0.081, -0.062) | <0.001 | -0.044 (-0.053, -0.036) | <0.001 |
| Unfavorable to gut microbiota | 1.116 (1.087, 1.146) | <0.001 | 1.116 (1.084, 1.148) | <0.001 | 0.045 (0.038, 0.052) | <0.001 | 0.038 (0.031, 0.046) | <0.001 |

Abbreviations: CI, Confidence interval; DI-GM, dietary index for gut microbiota; NHANES, National Health and Nutrition Examination Survey; OR, Odd Ratio; PIR, poverty income ratio.

^a^. The crude model was not adjusted for any covariates, while the adjusted model was adjusted for age, sex, race/ethnicity, marital status, education level, PIR, physical activity total MET, take anti-infectives drugs.

^b^. The DI-GM ranges from 0-11 (including beneficial to gut microbiota [ranges from 0-7] and unfavorable to gut microbiota [ranges from 0-4])

^c^. The frailty score is assessed as a continuous variable involving 49 questions related to multiple systems, with values from 0 to 1 assigned based on the severity of each question, and the sum of these scores is divided by the number of questions to result in a total score that ranges from 0 to 1.

^d^. Frailty was categorized as "yes" (≥0.25 scores) or "no" (<0.25 scores) according to Frailty score

**Supplementary Table 4. Threshold effect analysis of DI_GM and Frailty of the NHANES 2007-2020 March participants**

|  | **Adjusted OR (95% Cl)** | ***P*-value** |
| --- | --- | --- |
| Fitting model by standard linear regression | 0.917(0.893-0.941) | <0.001 |
| Fitting model by two-piecewise linear regression |  |  |
| Inflection point | 3 |  |
| DI_GM <3 | 1.04(0.935-1.161) | 0.472 |
| DI_GM >3 | 1.04(0.935-1.161) | <0.001 |
| *P* for likelihood ratio test | 0.016 | 0.016 |

Abbreviations: CI, Confidence interval; DI-GM, dietary index for gut microbiota

Model was adjusted for age, sex, race/ethnicity, marital status, education level, PIR, physical activity total MET, take anti-infectives drugs.
